# Lord Knutsford's Reply to "Ierne's" Shylock's Article

**Published:** 1918-06-22

**Authors:** 


### THE ECONOMICS OF NURSING.

### Lord Knutsford's Reply to "Ierne's" Shylock's Article.

MY answer to "Ierne" was not evasive. I replied, and reply, that nurses do not "above all other workers forfeit their freedom," unless it is called a forfeiture of freedom, which apparently "Ierne" so thinks it, to enter voluntarily into an agreement the terms of which are known before it is entered into. To compare this to "slavery," where there is no agreement, nor anything done voluntarily, is, I repeat, ridiculous. But let all this pass.

"Ierne" compares me to Shylock. Amen. I would rather have been Shylock than have sneaked out of my bargain, as the other man did, whose name I forget. "Ierne" may have the last word on the "frills" of the matter. All that is important is that nurses should generally all over the country be treated fairly. "Ierne" pins her demand to one particular reform—the one day in seven. In passing, I rather doubt whether an increase of twenty nurses to a staff of 140, as she writes, would give this. There are many things to consider. A probationer cannot, for instance, take a staff nurse's duty; therefore, adding twenty might not make the alteration possible.

I think "Ierne" overlooks, too, the great difficulties of providing the necessary increase of accommodation. In the last few years we have made very great changes for the better in the nurses' hours at the London Hospital. The additional cost in salaries has, of course, been great, and, moreover, it has necessitated building two nurses' homes, at a cost of £87,000, to accommodate the extra nurses. I do not say that all this was due to the better hours given to our nurses, but we could not have given these hours without building these two homes. Last year the nurses' salaries were increased by £4,000 a year. Pity for Shylock, please, who has to get the money!

"Ierne" is good enough to write that what I say to-day other hospital chairmen and boards will say to-morrow. I should be very proud if I thought this were even remotely true. I fear the opposite is more likely. My experience is that people generally say, "Oh, that's all very well for the 'London'—it is a rich hospital"; whereas it is the poorest, by far, of any of the big hospitals. But let me give the hours at the "London," and then, if "Ierne" is right, possibly they may be adopted at hospitals where the time off is less. Reforms cannot be carried in a day. They must be carried step by step. We have not, I hope, come to the end at the London Hospital yet, but whatever we do some people will ask for more, and not be happy till they get it.

It is instructive, however, when dealing with "hours off" to note that the hours off which we give at the "London" actually entail a *daily average of* 119 *nurses* off duty and away on holidays, days off, and sick leave, apart from those off for their three hours every day.

Here are the hours off:—

SISTERS.

Two hours off every day, in addition to meal-times.
Alternate Sundays off.
Half a day a week. So that every other week a sister gets from 2 P.M. Saturday to 11 P.M. Sunday.
One month's holiday a year (full pay).

NURSES.

Three hours off every day, in addition to meal-times.
One Sunday a month.
Half a day a week, which can be taken with the Sunday.
One month's holiday a year (full pay).

PROBATIONERS (DAY).

Three hours off every day, in addition to meal-times.
One day off a fortnight.

PROBATIONERS (NIGHT).

Three hours off every day.
One night off every fortnight. May sleep away—*i.e.* 10 A.M. till 1 P.M. the following day.
All probationers get a month's holiday every year (full pay).

NURSES ON THE PRIVATE STAFF.

The "days off" are, so to speak, saved up for them while at their cases, and accumulate at the rate of one day off for each fourteen days at a case. So that if a nurse is three months at a case she gets a whole week off on her return.

One month's holiday a year (full pay).

In addition to the above, all sisters, nurses, and probationers get almost indefinite sick leave on full pay. I have known it given for eighteen months.

As to number of nurses to patients, the following figures may be interesting. I take 1916 as the last figures I have at hand. The daily average number of patients was 812. These were nursed by 329 day nurses = 2.46 patients per nurse, and by 130 night nurses = 6.24 patients per nurse, or, taking the figures together, 812 patients were nursed by 459 nurses, which gives 1.77 patients per nurse. This assumes that the whole nursing staff was on duty, but see the paragraph as to the average number of nurses away each day.

NOW AS TO PAY.

Probationers get £17 the first year.
£24 the second.
If they become staff nurses, £30 the third year.
If they become holiday sisters, £40 the third year.
£45 the fourth, rising to £50 the next year if they stay in the service of the hospital.
If she joins the private staff after the second year she receives £35 for her third year, rising by £5 yearly to £50, with washing and everything found, including about £4 for uniform.
All nurses get 2s. 6d. a week for washing.
After six years, from the date of entering the hospital, every member of the staff gets an addition of £5 per annum to her salary. After twelve years a second addition of £5 a year is given. And after eighteen years' service all members of the staff on leaving get a full-pay pension for life calculated on their average earnings during the last three years of their service.

*Pace* "Ierne," who writes that "nurses work at a lower wage than her fellows similarly equipped." I do not think there are many professions open to women, even of the varied social planes from which nurses are recruited, who can say they have better terms.

KNUTSFORD.

